# Conservation Biological Control of Pests in the Molecular Era: New Opportunities to Address Old Constraints

**DOI:** 10.3389/fpls.2015.01255

**Published:** 2016-01-12

**Authors:** Geoff M. Gurr, Minsheng You

**Affiliations:** ^1^Institute of Applied Ecology, Fujian Agriculture and Forestry UniversityFuzhou, China; ^2^Fujian-Taiwan Joint Centre for Ecological Control of Crop Pests, Fujian Agriculture and Forestry UniversityFuzhou, China; ^3^Key Laboratory of Integrated Pest Management for Fujian-Taiwan Crops, Ministry of AgricultureFuzhou, China; ^4^Graham Centre, Charles Sturt UniversityOrange, NSW, Australia

**Keywords:** CRISPR/Cas9, gene drive, barcoding, gut analysis, gene editing, induced plant defense

## Abstract

Biological control has long been considered a potential alternative to pesticidal strategies for pest management but its impact and level of use globally remain modest and inconsistent. A rapidly expanding range of molecular – particularly DNA-related – techniques is currently revolutionizing many life sciences. This review identifies a series of constraints on the development and uptake of conservation biological control and considers the contemporary and likely future influence of molecular methods on these constraints. Molecular approaches are now often used to complement morphological taxonomic methods for the identification and study of biological control agents including microbes. A succession of molecular techniques has been applied to ‘who eats whom’ questions in food-web ecology. Polymerase chain reaction (PCR) approaches have largely superseded immunological approaches such as enzyme-linked immunosorbent assay (ELISA) and now – in turn – are being overtaken by next generation sequencing (NGS)-based approaches that offer unparalleled power at a rapidly diminishing cost. There is scope also to use molecular techniques to manipulate biological control agents, which will be accelerated with the advent of gene editing tools, the CRISPR/Cas9 system in particular. Gene editing tools also offer unparalleled power to both elucidate and manipulate plant defense mechanisms including those that involve natural enemy attraction to attacked plants. Rapid advances in technology will allow the development of still more novel pest management options for which uptake is likely to be limited chiefly by regulatory hurdles.

## Introduction

Developing more effective ways to manage agricultural pests using their natural enemies, ‘biological control,’ is critical for three interacting reasons. First, insect pests continue to cause severe damage to crops globally, estimated to be at least $470 billion per annum (Culliney, 2014). Second, the burgeoning human population, combined with increasing *per capita* demands for protein, require that losses from pests and other causes (including human wastage) are addressed so that food availability is doubled in coming decades (Tilman et al., 2011; Godfray and Garnett, 2014). Third, the availability of insecticides is threatened by the declining availability of products as a result of resistance in pest populations rendering them ineffective, as well as increasingly stringent regulatory demands for human and environmental safety. Accordingly, agricultural intensification efforts should be based on ecological principles rather than greater reliance on non-renewable resources (Bommarco et al., 2013). Such ecological intensification should enhance the contribution of ecosystem services, such as those provided by natural enemies that can suppress pest populations in place of insecticides.

In this review we briefly describe the history of the major forms of biological control to explain our focus on conservation biological control. We then go on to identify five particular constraints to the greater use of conservation biological control and for which molecular approaches are being employed, or potentially can help solve (**Table 1**). In the final section we review the relevant technologies.

**Table 1:** Major constraints to the impact of conservation biological control and how molecular techniques have contributed or might contribute (see text for full explanation including definition of acronyms).

Constraint	Technological contribution	Actual or potential/extent of use
Elucidating the identity of natural enemies in a system	PCR-based identification of natural enemies including microbes	Actual (e.g., Gariepy et al., 2014; Zhou et al., 2014; Duman et al., 2015)
Understanding who eats whom	Monoclonal antibody-based identification of natural enemies including microbes	Largely superseded (e.g., Calder et al., 2005)
	PCR- based tools for same	Actual, common (e.g., Symondson, 2002; Sheppard and Harwood, 2005)
	NGS-based tools for same	Actual but largely potential (e.g., Gomez-Polo et al., 2015; Piñol et al., 2015)
Inadequacy of specific agents	Genetic modification of natural enemies to provide improved traits	Actual for microbial agents (e.g., Sun et al., 2004; Yamamoto-Tamura et al., 2011; Ryder et al., 2012; Bonaldi et al., 2015). Largely potential for arthropod agents (but see Hoy, 2000)
Mortality of natural enemies from widespread pesticide use	Widespread use of genetically modified crops expressing insecticidal toxins, so allowing large reductions in insecticide use thereby promoting regional natural enemy populations	Actual (e.g., Lu et al., 2012).
Slow or limited movement of natural enemies from donor habitat to a crop attacked by pests	Genetic modification of crop plants to respond more quickly and strongly to pest attack with volatile signals attractive to natural enemies.	Largely potential (but see Bruce et al., 2015)

## Biological Control: Development of the Discipline and Constraints to its Impact

Biological pest control by predators and parasitoids, as well as entomopathogenic nematodes and microbes, has been in action since the dawn of agriculture and there is a long history of farmers seeking to increase its efficacy. Ant colonies have been traded and the movement of ants between tree crop canopies facilitated by bamboo poles for 100s of years (van Lenteren, 2012). Since the 1800s, ‘classical biological control’ efforts to suppress exotic pests have involved importing their natural enemies; though this practice is increasingly under scrutiny to avoid effects of introduced agents on non-target, native species (Follett and Duan, 2012). During the 1900s, culturing methods were developed for many species of natural enemy, allowing inundative biological control programs against a range of pest targets (van Lenteren, 2012). This practice continues to grow in popularity though the costs to farmers are often significantly higher than the use of insecticide applications.

Conservation biological control is distinct from the preceding forms in that it involves making better use of agent species that are already present in a region rather than importing or mass releasing new species. A major way to achieve this is by habitat management in which vegetation patterns and farming practices are revised to provide key ecological resources to natural enemies or to have direct effects on pests independent of natural enemies (Poveda et al., 2008; Letourneau et al., 2011; Lu et al., 2014). Herbivorous arthropods are generally favored in agricultural systems by monocultures of highly preferred host plants and paucity of natural enemies. Accordingly, conservation biological control often seeks to suppress pests by strategic diversification of vegetation to encourage the activity of predators and parasitoids (Landis et al., 2000; Knight and Gurr, 2007). The discipline of conservation biological control has evolved rapidly over the last two decades from an often hit-and-miss exercise, involving for example, the well intentioned sowing of seed mixes containing multiple species of plant for which there was little of no information on their benefit to natural enemies and possible use by pests. The term ecological engineering was introduced into the pest management arena to reflect a more rigorous approach to conservation biological control in which the intervention is more explicitly supported by theoretical and empirical evidence to maximize the likelihood of impact and adoption (Gurr et al., 2004). A key issue in the use of this more reductionist approach to conservation biological control is deciding which natural enemy species to focus on and avoiding misidentification or failure to recognize cryptic (e.g., sister) species. Levels of taxonomic knowledge of natural enemy are low in many systems, especially for microbial and nematode agents and this is one constraint where molecular techniques are increasingly applied (**Table 1**).

The development of conservation biological control programs is driven strongly by empiricism. It can involve laboratory studies of the species of flowers that are attractive to and best support parasitoids (Zhu et al., 2013) and predators (Zhu et al., 2014). Some studies also aim to identify plant species that are used by pests – either as adults (Zhu et al., 2015) or as larvae (Begum et al., 2006) – so that these can be avoided in later field efforts. In cases where farmer uptake has been strong, such as the use of a ‘push-pull’ system in east Africa (Khan et al., 2000), careful laboratory studies have been important in plant species choice. Chemical analyses and behavioral studies with parasitoids were essential in identifying the fact that genotypes of molasses grass (*Melinis minutiflora* P. Beauv.) differed widely, with some producing much more of the parasitoid foraging attractant nonatriene whilst others produced such low levels that no significant parasitoid response was evident (Khan et al., 2000). Optimal genotypes of this plant were then selected for use as an intercrop plant to draw parasitoids into the crop. Consistent with this example, field experimentation and on-farm adoption are now often preceded by rigorous laboratory experimentation to identify optimal types of diversity that selectively benefit the natural enemy more than the pest. Recent quantitative syntheses of vegetation diversification schemes in experimental evaluation lend strong support for the potential of these methods to suppress pests, promote natural enemies and to reduce crop damage (Letourneau et al., 2011). Uptake in farming systems has occurred in various parts of the world including Asia, Africa, Europe, the Americas and Australasia but at a global level is very low. Only one scheme we are aware of – the push-pull system mentioned above – is widely adopted across multiple countries (Khan et al., 2000). Accordingly, there is a need to develop better and more widely applicable strategies that will allow the promise of conservation biological control to be realized more widely in global agriculture. Key to achieving this objective is the capacity to untangle the often complex food webs in agricultural systems where a given predator may consume multiple species of prey in addition to plant foods (e.g., pollen). Even simpler issues such as which herbivore individuals are attacked by a given parasitoid can be challenging using conventional techniques based on rearing, culturing and *in vitro* identifications. The capacity of molecular approaches to address this is great (**Table 1**).

Ecologists have identified that vegetation patterns at the landscape scale – not only local scale - strongly affect local impact of natural enemies (Perovic et al., 2010; Paredes et al., 2015). Work on landscape-scale effects in biological control is currently very active and starting to move from a dominance of empirical studies to the development of a stronger theoretical foundation and the formulation of hypotheses to explain the responses of arthropods to vegetation patterns. A key constraint to the use of landscape-scale approaches to be actively exploited in conservation biological control is that agricultural landscapes are often treated heavily with insecticides that can greatly impact natural enemies. Molecular methods can address this issue by genetic modification of agents to make them less susceptible to particular insecticide types, or to impart other desired traits (**Table 1**).

Local-scale and wider-scale effects can interact strongly and offer scope for manipulation. This is reflected in recent work that has sought to exploit the induced plant defense mechanism based on herbivore-induced plant volatiles (HIPVs) that attract predators and parasitoids to attacked plants. Manipulating this form of plant defense by the application to crops of exogenous forms of HIPV, or compounds that trigger endogenous HIPV production, can draw natural enemies from nearby donor habitat into crops when pests arrive (James, 2003, 2005; James and Price, 2004; James and Grasswitz, 2005; Mallinger et al., 2011). Some such work has also involved rewarding natural enemies on arrival with groundcover or crop-margin plants that produce nectar as a supplementary food (Simpson et al., 2011a). In this general approach to conservation biological control there are particularly exciting opportunities for molecular techniques to be applied based on gene editing tools to elucidate the mechanisms underlying this form of plant defense, and to enable the production of plants with an enhanced capacity to attract biological control agents (**Table 1**).

## Oppourtunities for Use of Molecular Technologies

In this section we consider the extent to which molecular technologies have already contributed to the development of biological control with an emphasis on conservation biological control. Within each of these sections we look ahead to emerging molecular – and especially DNA-based technologies – to highlight what we see as particularly exciting opportunities for conservation biological control to be made more powerful.

### Elucidating the Identity of Natural Enemies in a System

Accurate identification of agent species is critical in all branches of biological control. For example, when a new, invasive pest species becomes the target of a biological control program, a molecular diagnostic tool is a powerful way to elucidate which parasitoids attack the pest and might be the focus of further investigation (Gariepy et al., 2014). More generally, the accurate identification of parasitoids is a critical initial step in considering their suitability as biological control agents. For example, work seeking to determine the parasitoid fauna of soybean aphid (*Aphis glycines* Matsumura) in China complemented preliminary morphological identification with DNA barcoding to generate DNA sequence data for the mitochondrial cytochrome c oxidase subunit I (COI) gene and the D2 region of 28S rDNA (Zhou et al., 2014). Equivalent work has focused on sunn hemp (*Crotalaria juncea* (L.)) pest parasitoids using the COI gene (Duman et al., 2015). The standard approach in barcoding involves extracting DNA from a sample and – using appropriate primers – amplify a DNA region such as the CO1 gene after which the amplicons are sequenced, then compared to the barcode of life database (BOLD; Ratnasingham and Hebert, 2007) where the level of similarity is matched with earlier accessions of known identity which – at the time of writing – is approaching 4.5 million.

In conservation biological control, however, taxonomy is a particular challenge because an intervention – such as establishing a strip of nectar plants in the border of a crop – might have an effect on many species of predator and parasitoid and even be used by species at other trophic levels: herbivores and hyper-parasitoids. Other conservation biological control approaches, especially those in which plant chemical ecology is manipulated, may also have effects on microbes and nematodes including entomopathogenic forms, largely in the below-ground environment where identification presents additional challenges associated with the low level of described species and relative unavailability of specialist taxonomists. Traditional taxonomic approaches based on morphological characters, and biochemical properties for some microbes, also tend to be laborious, even if the fauna of a region is well described. For this reason, taxonomists have rapidly adopted DNA-based techniques for identifying species. DNA ‘barcoding’ has become a routine procedure for the identification of individual organisms in biological control studies where it offers the distinct advantage of allowing the identification of life stages such as eggs, immatures and males that can be otherwise impossible to identify (Lima et al., 2008). Significantly, though other forms of biological control are usually concerned with identifying a single agent species or discriminating between a few related forms using a small number of reference specimens, conservation biological control often needs to grapple with the identification of many individuals of multiple species not all of which are even tentatively identified. Here, DNA barcoding offers great power because a given individual or sample can be subjected to barcoding and the sequences from each species identified against a reference database. This allows species level identifications from a sample even where there is a complete lack of taxonomy; a capacity that is growing rapidly with the increasing numbers of named accessions.

### Understanding Who Eats Whom

The capacity of molecular techniques to provide species level identification even for fragmentary remains of a species lends them great capacity in the area of analyzing the gut contents of predatory biological control agents. The power of the technique extends to the identification of liquid prey remains and this is essential in studies of suctorial feeders such as spiders, mites and predatory insects such as Hemiptera and Thysanoptera. The method also lends itself to studying the remains of plant meals in the gut of omnivores (Pumariño et al., 2011). These advantages of molecular identification over observational approaches and attempting to identify fragmentary remains in the gut visually have revolutionized the study of predator-prey relationships and wider food web links. Indeed, elucidating trophic linkages has become one of the most active areas at the interface of biological control and molecular techniques. The past 15 years has seen this important field move from use of monoclonal antibodies to PCR-based approaches (Symondson, 2002; Sheppard and Harwood, 2005). The approach is essentially the same as that employed for identifying biological control agents described above and has been employed in many studies of diet range in biological control and wider aspects of ecology in multiple taxa (Symondson, 2002). A constraint to the method, however, has been the relatively high costs of reagents and labor. Whilst this is not a major issue in simple studies of biological control agent identity, a given predator may contain DNA from multiple prey species, necessitating many assays. Further, many individual predators need to be processed in order to obtain a meaningful picture of predation patterns from field-sampled predators. Thus, targeted barcoding assays using single round or multiplex PCR are appropriate for the study of stenophagous predators in which a limited range of prey species is found but less suitable in other cases. For these reasons, next generation sequencing (NGS)- approaches are starting to be embraced in cases where there is a need to screen for the presence of multiple prey species and where only limited information is available on the identity of diet components.

In DNA metabarcoding, direct characterization of multiple samples with several thousand sequences is possible for each PCR product, so giving the capacity to reveal the consumption of multiple prey species simultaneously (Pompanon et al., 2012; Clare, 2014; Symondson and Harwood, 2014). Metabarcoding represents the current frontier of molecular diet studies and its power has recently been demonstrated in a study of an omnivorous vertebrate, the brown bear (De Barba et al., 2014). Application in biological control studies is at a very early stage but likely to expand, reflecting the fact that the equipment necessary for NGS approaches is becoming more widely available and kit-based platforms are reducing the levels of training required. A particular need, however, is to integrate the results from NGS with ecological methods (Furlong, 2015). An example of such integration is Spanish work on lettuce pests in which NGS was used to study the diet to two major predators (Gomez-Polo et al., 2015). The value of the NGS approach was that it enabled the detection of intra-guild predation, a phenomenon not evident in concurrent PCR assays. The predator *Orius majusculus* (Reuter) was found to prey on other species of *Orius*, syrphids, linyphiid spiders, and predatory labybird beetles as well as on unidentified Diptera and Lepidoptera. This example illustrates the capacity of NGS for untargeted screening that can uncover unexpected trophic relationships and levels of complexity.

Declining costs resulting from economies of scale and competition between suppliers will facilitate further adoption of DNA technologies for unraveling biological control food webs. Naturally, technical challenges remain. For example, variable primer-template mismatches of species within a sample hamper the use of high-throughput DNA sequencing. The extent of this was explored in a recent study using an artificially constructed community of 12 species of terrestrial arthropods and suggested that the potential of the technique to provide quantitative information is limited (Piñol et al., 2015). Accordingly, only the qualitative results – that is the list of prey species recorded from each predator – can be relied upon. In this respect, however, high-throughput DNA sequencing is equivalent to other, simpler, molecular approaches that yield only qualitative results such that a given predator is either positive or negative to the remains of a given prey species (Greenstone et al., 2014). Even a non-quantitative knowledge of the identity of prey species consumed by a given predator individual can allow a ‘pragmatic and useful surrogate for truly quantitative information’ (Symondson and Harwood, 2014) to be assembled from field captured insects; illustrating, for example, the habitats or times of year in which a given predator is most likely to exploit a given prey. Accordingly, despite the methodological limitations of molecular methods, they have demonstrated value to demonstrate phenomena such as the significance of certain generalist predators in restricting the escalation of pest numbers early in the crop season before the arrival of more specialized enemies such as parasitoids. Importantly, however, if the results from assays of field-captured predators are complemented with laboratory studies to determine the relative detectability periods for prey DNA for each possible combination of predator and prey, it becomes possible to generate valid quantitative data to determine the relative importance of each trophic link. The collection of papers in the special issue edited by Symondson and Harwood (2014) constitutes a detailed picture of the state of this field and technical challenges ahead. Further advances in technology will help provide a fuller understanding of poorly understood areas of complexity such as the role of nematode and microbial natural enemies in food webs and the interplay between above- and below-ground effects (Fisher et al., 2011).

### Addressing the Inadequacies of Specific Agents

Most work with genetically modified biological control agents has focused on microbial agents (Sun et al., 2004; Yamamoto-Tamura et al., 2011; Ryder et al., 2012; Bonaldi et al., 2015). But the capacity to modify the genome of insects has been a reality since the end of the last century (Fraser, 2012). Indeed the first field trial of a genetically modified arthropod biological control agent was with the predatory mite *Metaseiulus occidentalis* in 1996 (Hoy, 2000). Relatively little work followed that, despite the obvious advantages to biological control of conferring a trait such as insecticide resistance; allowing the agent to be used even in settings where the target pest’s density, or presence of additional pests, required the use of insecticides. Computer modeling of the dispersal of an insecticide (azinphosmethyl) resistant walnut aphid parasite, *Trioxys pallidus* (Caprio et al., 1991) indicated that the establishment of the resistant strain to the extent that it comprised half of the population in at least 90% of Californian orchards would take 5–7 years. This relatively long time period may partly explain the lack of use of the method; compounded by factors such as the fact that transgenes may be rapidly lost under field conditions because of fitness costs (Hoy, 2000). Amongst the traits that might be usefully engineered into a biological control agent is a ‘kill switch’ based on extreme susceptibility to a spray-on compound so that it could be exterminated if it became a pest or had unexpected off-target impact.

The wider potential of manipulating the traits of biological control agents – though not necessarily by trangenesis – is evident in work on the attraction of entomopathogeninc nematodes to the volatile signals of maize infested by pests (Hiltpold et al., 2010). *(E)*-beta-caryophyllene is weakly attractive to *Heterorhabditis bacteriophora* but just six generations of selection led to a strain of the nematode that responded more strongly, moving twice as rapidly compared with the original strain. Field evaluation demonstrated that the selected strain was significantly more effective at reducing populations of the pest provided that the maize cultivar was one capable of emitting *(E)*-beta-caryophyllene when under attack. For maize varieties that did not produce this semiochemical from its roots, there was no such response by the nematode. This study illustrates the potential interplay between the agent and the crop plant, both of which potentially can be manipulated to promote biological control. Scope for doing this by crop plant manipulation is covered below.

Prospects for the wider use of genetically modified biological control agents are strong given the very recent advent of the gene editing tool clustered regularly interspaced short palindromic repeats (CRISPR) which is much cheaper and less technically challenging than earlier approaches such as transcription activator-like effector nucleases (TALENs) and zinc finger nucleases (Doudna and Charpentier, 2014). Reflecting the rapidity of technological developments in DNA techniques, a relatively recent major review of insect transgenesis (Fraser, 2012) did not mention CRISPR/Cas9. More recent articles indicate that applied scientists are anticipating its use for control of invasive species (Webber et al., 2015) and mosquitoes (Hall et al., 2015). This gene editing technology offers scope to make very precise changes in genomes with the insect’s own genes being up- or down-regulated to the benefit of pest management. This may also reduce the fitness costs that tend to be associated with arthropods genetically modified by older approaches involving transgenesis of genetic material from other species. If coupled with gene drive (Webber et al., 2015), a modified insect could rapidly establish and even replace the wild type. A deleterious trait engineered into a pest population in its invaded range could flow to the species in its geographical origin, where it is not considered a pest and may be a valuable component of biodiversity, resulting in global extinction. Given this great power, current use is confined to the laboratory where stringent confinement measures are taken (Akbari et al., 2015). Eventually, however, the technology will need to be evaluated under field conditions and commentators fear that the risks of spreading a deleterious gene widely in a pest population may limit use – to islands for example (Webber et al., 2015). A less risky alternative is to employ gene drive to biological control agent species – such as the predators and parasitoids of pests – rather than to the pest species themselves. In this instance, the trait that is spread through the agent species would confer an advantageous rather than deleterious trait. The major advantage of this approach is that there is no risk of irreversible extinction of the modified species because an advantageous trait is used. Traits that could be engineered into an agent species might include the capacity to cope with the higher temperatures resulting from climate change (Wilson et al., 2015) or to tolerate exposure to pesticides in sprayed crops (Hoy, 2000). Agents might also be engineered to respond more effectively to the chemical signals released by plants when attacked by a pest, to (*E*)-beta-caryophyllene for example (Hiltpold et al., 2010). Gene drive allows rapid spread in the population of such agents the capacity to detect and respond to plant cues. Used to manipulate biological control agent rather than pest targets, gene drive presents no risk of extinction of the manipulated species though there is a theoretical risk that a ‘super-agent’ could disperse to a target species’ point of origin and have unwanted impact. This risk is manageable, however, if the pest system and introduced traits are appropriate.

To date, the most active field of genetic modification to support insect pest management is with the use of paratransgenic species. These are not, themselves, subject to genetic modification but carry microbial symbionts that are modified. Genes expressed by these microbes, most commonly located in the insect gut, affect the biology of the host insect. This method has been pursued most commonly to manipulate malarial mosquitoes (Sassera et al., 2013; Sharma et al., 2013; Wilke and Marrelli, 2015) and other vectors of diseases including tsetse flies, human lice, and triatomine bugs (Medlock et al., 2013; Wamwiri et al., 2014; Taracena et al., 2015). The method has also begun to be explored to control the Asian citrus psyllid (*Diaphorina citri*) Kuwayama, the vector of the citrus pathogen Candidatus Liberibacter spp. Broadly equivalent work has been reported with the planthopper, *Perkinsiella saccharicida*, for Fiji leaf gall control (Hughes et al., 2011) and the glassywinged sharpshooter (*Homaldica vitriapennis*) which transmits the bacterium *Xylela fastidiosa* responsible for various crop diseases including Pierce’s disease (Ramirez et al., 2008). Whilst the foregoing cases of paratransgenic species use are not examples of conservation biological control, they illustrate the potential for this approach to be used in a range of insect taxa and application to biological control agents is likely to follow.

### Reducing Pesticide-Induced Mortality of Natural Enemies

Over many decades, a large volume of literature has established that many pesticides cause lethal and sub-lethal adverse effects on natural enemies and this is clearly detrimental to effective biological control. One potential solution to this, as discussed above, is the development of insecticide tolerant strains of agent. Progress on that front has been limited but a second aspect of molecular biology has led to widespread effects. The advent of genetically modified crops expressing insecticidal toxins such as those from *Bacillus thuringiensis*, has led to major reductions in the amounts of pesticides applied per unit area. Since some of these crops, especially cotton and maize, are now grown over extensive areas in many counties, this has led reductions in pesticide use at the regional scale. Studies of this phenomenon in China shown increased densities of predatory insects and spiders in these GM crops. Importantly, these biological control agents spillover from GM cotton into neighboring, non-GM crops (Lu et al., 2012). This creates important opportunities for conservation biological control because it enhances the amount of donor habitat in the landscape from which natural enemies may colonize other crops, particularly those in which local scale manipulations have been made by altering the within-crop vegetation or by enhancing the capacity of the crop plants to attract ‘bodyguards’ with HIPVs. A caveat to the foregoing argument is that there is evidence that the declines in insecticide use in Bt crops have led to a resurgence of sucking pests such as true bugs that are unaffected by Bt toxins. Accordingly, populations of these secondary pests may build up, and spillover from, GM crops (Lu et al., 2010).

A further, potential use of GM crops in conservation biological control is to employ them as dead-end trap crops to which gravid pests are attracted, leaving the focal crop less severely infested (Keeping et al., 2007). Accordingly, the rise of GM crops presents opportunities for conservation biological control to promote pest suppression and these outweigh risks to natural enemies from the effects of gene products. Though some studies have shown genetically modified crops can lead to adverse effects on natural enemies, that finding is based on comparisons with non-GM crops that are unsprayed. A more realistic comparison is with non-GM crops that are treated with insecticide. Under these conditions, the risks to natural enemies of GM crops are lower than the risks from insecticide use (Gatehouse et al., 2011). Many studies, however, report no effect of GM crop traits on natural enemies (García et al., 2012; Silva et al., 2014).

### Promoting Movement of Natural Enemies from Donor Habitat

Work over the last decade has sought to exploit chenical ecology in biological control. Synthetic HIPVs are applied to the crop or released from dispensers placed within the crop to attract natural enemies from nearby donor habitat (James, 2003, 2005; James and Price, 2004; James and Grasswitz, 2005; Mallinger et al., 2011). A recent extension of this idea, the attract and reward strategy (Simpson et al., 2011a,b; Orre-Gordon et al., 2013), provides food and shelter resources in the form of nectar-bearing plants to enhance the survival and longevity of the attracted predators and parasitoids (Hatano et al., 2008; Simpson et al., 2011a; Orre-Gordon et al., 2013). It is likely that the effect of exogenous HIPVs on natural enemy attraction is not confined to volatilization of the applied compound since this dissipates rapidly. Accordingly the exogenous compounds likely elicit the endogenous production of HIPV blends by the treated plant and this phenomenon lends itself to genetic manipulation. The capacity to make even major changes to the volatiles produced by plants is evident in very recent work which produced a GM wheat line that secretes the volatile aphid alarm pheromone, (*E*)-β-farnesene which is used by parasitoids to detect patches of aphid hosts. Laboratory studies demonstrated repellence of three species of aphid and enhanced foraging by the parasitoid *Aphidius ervi* in response to volatiles from this wheat line (Bruce et al., 2015). Though initial field testing did not reveal enhanced parasitism or reduction in aphids, this example illustrates scope to engineer crop plants to synthesize novel or greater amounts of semiochemicals, opening possibilities for more effective ‘attract and reward’ or strategies in which ‘sentinel plants’ that are especially sensitive to a threat (e.g., pest attack) provide a volatile cue to the main crop that also has been engineered to respond strongly (Pickett et al., 2014). Sobhy et al. (2014) note the regulatory and public acceptance hurdles associated with the use of transgenic crops so favor the use of elicitors and ‘plant strengtheners’ to enhance biological control by activating or elevating the expression of existing genes, thereby circumventing the need for GM approaches. The very recent advent of CRISPR allows very precise changes in genomes so may reduce the barriers to regulation and adoption (Voytas and Gao, 2014). CRISPR/Cas9 certainly offers great scope to elucidate the genetic control of plant defenses in support of more powerful enhancements to biological control. Though scientific and regulatory issues mean that such genetic technologies are some way from commercial application, and they will remain an anathema to those who view conservation biological control as a cousin to organic agriculture, they likely represent part of the future toolbox.

## Conclusion

Molecular approaches offer much to the study and development of biological control but Chisholm et al. (2014) have emphasized the need for their use to be appropriate to the specific issue and for similar attention to be given to new statistical and digital approaches. This is consistent with the call by Furlong (2015) to use molecular methods for the study of trophic linkages together with ecological methods in the field. Of course the potential for molecular techniques to contribute to pest management is far wider than the conservation biological control issues dealt with this review. Other relevant examples include studies of pest genomes that can identify targets for novel toxicants or gene-based approaches based on gene drive. Also studies of key physiological processes such as digestion, immunity (Xia et al., 2015), insecticide detoxification (You et al., 2013), and thermotolerance (Ge et al., 2013) are being approached with molecular tools to provide unparalleled power to elucidate genetic control and transcriptional regulation. These too offer scope for developing novel pest management approaches. Certainly, rapid advances in technology will facilitate still more novel pest management options for which uptake is likely to be limited more by regulatory hurdles (Voytas and Gao, 2014) than technical obstacles. These hurdles include the differing types of systems in place across counties (for example, based on either the *process* used to create a genetically modified organism or the *product* itself and the inherent risk presented by its altered traits) as well as broader issues of public acceptance. In Europe, for example, transgenic crop varieties are not cultivated even if they are approved for wide scale use in the Americas or elsewhere. Ultimately, however, neither the molecular approaches nor conservation biological control that is enhanced by the use of molecular approaches will be a ‘silver bullet’ panacea for pest management. The appropriate and – wherever possible – synergistic combination of pest management tools (Knight and Gurr, 2007; Gurr and Kvedaras, 2010) will remain key to sustainability.

## Author Contribution

GMG and MY wrote the manuscript.

## Conflict of Interest Statement

The authors declare that the research was conducted in the absence of any commercial or financial relationships that could be construed as a potential conflict of interest.
